# Spontaneous atraumatic rupture of the urinary bladder following alcohol intoxication: A rare case report

**DOI:** 10.1002/ccr3.9395

**Published:** 2024-08-29

**Authors:** Shritik Devkota, Sugat Adhikari, Manbir Singh, Samiksha Lamichhane, Dipendra Adhikari, Bishal Koirala, Harsha Bhola

**Affiliations:** ^1^ Department of Radiodiagnosis and Imaging Anil Baghi Hospital Punjab India; ^2^ Shreegaun Primary Health Care Center Dang Nepal; ^3^ Department of Urology Anil Baghi Hospital Punjab India; ^4^ Department of Radiodiagnosis and Imaging B. P. Koirala Institute of Health Sciences Dharan Nepal; ^5^ Tuberculosis treatment center, Gandaki Province Pokhara Nepal; ^6^ Department of General Surgery Anil Baghi Hospital Punjab India

**Keywords:** alcohol intoxication, chronic alcoholic, intraperitoneal rupture, spontaneous rupture of the urinary bladder

## Abstract

**Key Clinical Message:**

Consideration of spontaneous urinary bladder rupture in the differential diagnosis of acute abdominal pain for alcohol‐abusing patients is crucial for ensuring timely surgical intervention and preventing life‐threatening complications due to its high associated morbidity and mortality.

**Abstract:**

Spontaneous rupture of the urinary bladder (SRUB) is a rare but critical urological emergency, typically associated with malignancy, neurogenic dysfunction, or previous radiation therapy. Here, we present a unique case of SRUB in a 65‐year‐old chronic alcoholic male who presented with acute lower abdominal pain following heavy alcohol consumption. Initial evaluations revealed leukocytosis, elevated serum creatinine levels, and ultrasound findings suggestive of bladder rupture. Computed tomography confirmed the diagnosis, indicating an intraperitoneal rupture with associated hematoma. Immediate surgical repair was performed, leading to a successful outcome. This case underscores the importance of considering SRUB in patients with acute abdominal pain, especially in the context of alcohol intoxication, and highlights the diagnostic and therapeutic challenges associated with this condition. Early recognition and intervention are crucial to prevent life‐threatening complications associated with urinary bladder rupture.

## 1 INTRODUCTION

Urinary bladder rupture, though infrequent, represents a critical urological emergency that can lead to substantial morbidity and mortality. Etiologically, bladder rupture can be classified as traumatic, spontaneous, or iatrogenic. Spontaneous bladder rupture is particularly rare and typically occurs in pathological bladders affected by malignancy, neurogenic dysfunctions, or following radiation therapy. Spontaneous rupture of the urinary bladder (SRUB) in individuals with chronic alcoholism is exceedingly rare but holds significant clinical importance due to the underlying pathophysiological mechanisms and potential complications. Chronic alcohol consumption can lead to chronic cystitis and increased bladder pressure due to overdistention, both of which may predispose the bladder to rupture without external trauma.^1^, ^2^ This report presents a unique case involving a 65‐year‐old male with a history of chronic alcoholism who was admitted to the hospital with severe lower abdominal pain. Initial examinations and diagnostic tests revealed a SRUB. This case is especially noteworthy as it highlights the occurrence of SRUB in the context of acute alcohol intoxication.

## 2 CASE PRESENTATION

### 2.1 Clinical history and clinical examination

A 65‐year‐old chronic alcoholic male, presented to the emergency department complaining of intense lower abdominal pain. He reported a history of heavy alcohol consumption over the past few days, including a binge drinking episode the night before. He and his attendants denied any recent trauma or fall. He explained that he had been experiencing nausea and vomiting over the past 24 h. On examination, he appeared distressed and mildly confused, with a blood pressure of 100/60 mmHg, a heart rate of 110 bpm, and a temperature of 37.8°C. Abdominal examination revealed tenderness in the lower abdomen, particularly in the suprapubic region, without guarding or rebound tenderness. The patient revealed the last episode of micturition to be 9 h back. Based on this, an initial provisional diagnosis of acute urinary retention was made and Foley's catheter was inserted. Following insertion, hematuria was seen on the urobag. Routine blood investigations and ultrasonography of the abdomen were ordered.

### 2.2 Investigations

**FIGURE 1:**
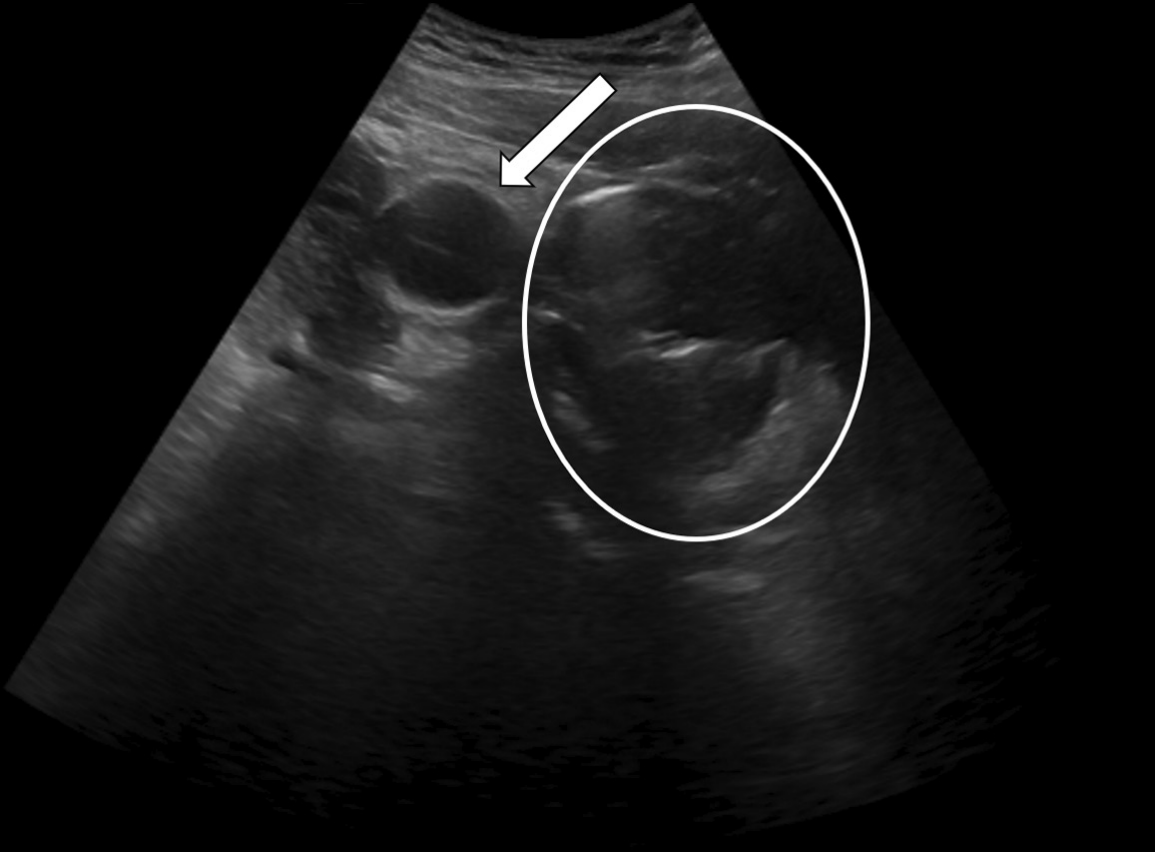
Ultrasonography image showing foley's bulb (white arrow) lying outside urinary bladder (white circle).

**FIGURE 2:**
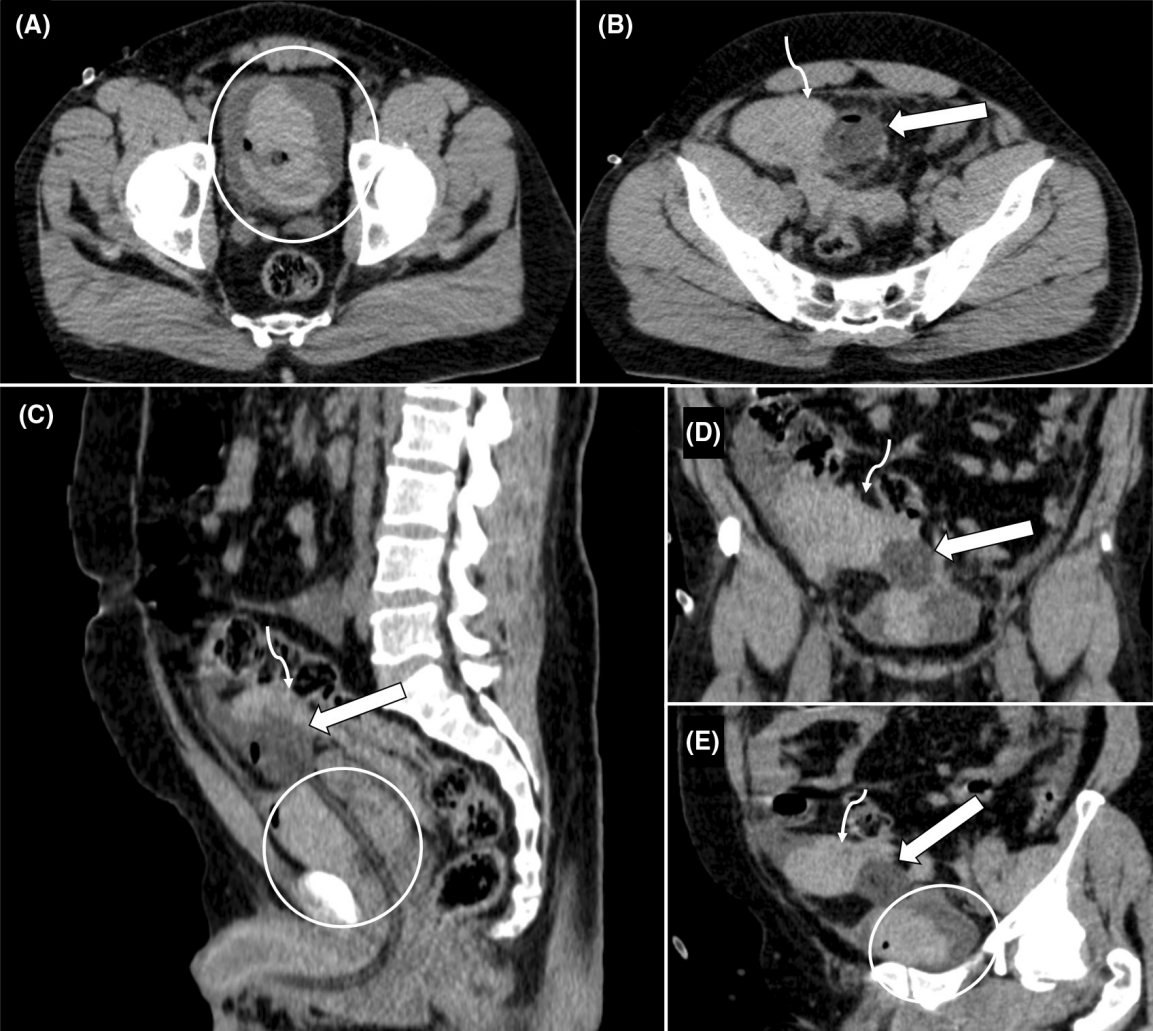
Axial (A, B), sagittal reconstructed (C), coronal reconstructed (D), and coronal oblique reconstructed (E) images of non‐contrast CT showing urinary bladder hematoma (white circles) with foley's bulb (white arrows) lying outside urinary bladder traversing through the dome and surrounding intraperitoneal hematoma (curved arrows).

Blood tests revealed leukocytosis (white blood cell count: 13,000/mm^3^) and elevated serum creatinine levels (1.5 mg/dL). The electrocardiogram showed sinus tachycardia without any ischemic changes. Bedside ultrasound (Figure 1) demonstrated a Foley bulb lying outside the urinary bladder, giving suspicion of rupture of the urinary bladder. The patient was shifted to the CT complex for confirmation of the ultrasound findings. NCCT scan of the pelvis (Figure 2) showed Foley's bulb lying outside the urinary bladder with the catheter traversing through the dome and surrounding intraperitoneal and intravesical hematoma suggesting intraperitoneal rupture.

Based on the clinical presentation and investigative findings, a diagnosis of SRUB was made, likely precipitated by acute alcohol intoxication.

### 2.3 Treatment

**FIGURE 3:**
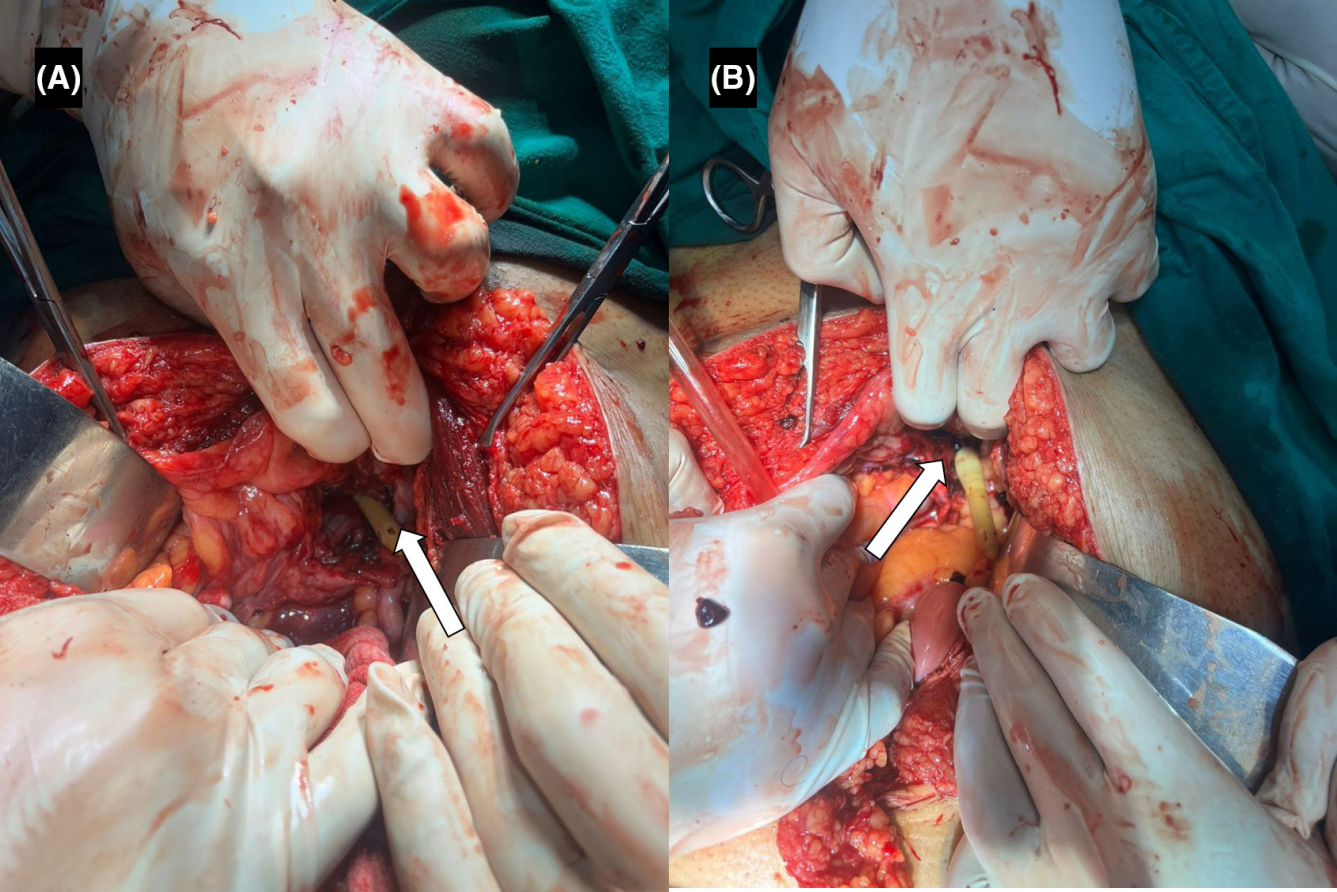
Intraoperative images (A, B) showing part of foley's catheter (white arrows) outside the urinary bladder with a defect in the dome of urinary bladder.

The patient was immediately started on intravenous fluids and broad‐spectrum antibiotics to prevent sepsis. Given the intraperitoneal nature of the rupture and the associated risk of complications such as peritonitis and abscess formation, emergent surgical repair was recommended. An exploratory laparotomy confirmed the diagnosis intraoperatively, revealing intraperitoneal blood‐mixed fluid. A rent (Figure 3) of approximately 4 cm was observed near the dome of the bladder, along with an intravesical hematoma. Clots were removed, and the bladder wall rent was repaired in two layers using 3‐0 Vicryl sutures. The procedure was uneventful.

### 2.4 Follow‐up

Postoperatively, the patient showed gradual improvement in both symptoms and laboratory parameters. He was discharged after a week of hospitalization. During follow‐up visits, he received counseling on the risks associated with alcohol abuse and was advised to seek support for alcohol cessation to prevent future complications.

## 3 DISCUSSION

In emergency scenarios, the urinary bladder can rupture due to various types of trauma or even spontaneously. Notably, there has been an increase in cases where healthy bladders rupture spontaneously, particularly following heavy drinking sessions. These instances are categorized under a specific condition known as idiopathic bladder ruptures.^3^ Bladder rupture, predominantly caused by trauma (96% of cases), is a rare occurrence with an incidence of about 1 in every 126,000 people. The rupture can be extraperitoneal (~60%–65% of cases) or intraperitoneal (25% of cases).^4^

SRUB has a high mortality rate despite being rare, due to delayed diagnosis. It is often overlooked and difficult to distinguish from other causes of acute abdominal pain, especially without a history of trauma.^5^ Bastable et al. as far back as 1959, documented a series of bladder rupture cases linked to alcohol intoxication, which had a mortality rate of 50%. Festini and team reported a 12% mortality rate for spontaneous intraperitoneal bladder ruptures occurring during episodes of excessive alcohol consumption.^6^, ^7^ Alcohol‐impaired patients present diagnostic and therapeutic challenges due to unclear medical histories and vague symptoms, potentially masking conditions from mild to severe, with delayed disease progression and untreated bladder rupture carrying high mortality risks.^8^, ^9^, ^10^

SRUB is along linked to malignant disease, bladder obstruction, neurogenic bladder, substance abuse, pelvic irradiation, and conditions weakening the bladder walls, with only handful of cases attributed to alcohol intoxication. Intraperitoneal ruptures often involve the bladder's dome.^10^, ^11^, ^12^, ^13^, ^14^, ^15^ In the early stages, bladder rupture symptoms can be minimal and vague, often leading to delayed diagnosis and confusion with other abdominal conditions. Therefore, high level of clinical suspicion is required for diagnosis of SURB. Early diagnosis of non‐traumatic SRUB is crucial for positive outcomes.^5^, ^8^ Patients with extraperitoneal rupture typically experience abdominal pain, distension, urination difficulties, and fever. In contrast, intraperitoneal rupture presents with macroscopic hematuria, abdominal pain, and voiding difficulties. Intraperitoneal SRUB, which often results in urinary ascites and abdominal infections, presents more severe symptoms than extraperitoneal SRUB. As the condition progresses, atypical symptoms may arise, making a detailed follow‐up history essential for diagnosis, especially in cases involving recent heavy alcohol consumption, abnormal behavioral response, altered bladder fullness, and urinary retention.^14^, ^15^, ^16^

Complications from this condition can lead to intra‐abdominal and pelvic abscesses, sepsis, and metabolic imbalances. The peritoneal reabsorption of significant amounts of urea and creatinine can give the appearance of acute renal failure in initial biochemical tests. As leaked urine is reabsorbed in the peritoneal cavity, it may cause electrolyte imbalances such as hyperkalemia, hypernatremia, uremia, or acidosis. Hyperkalemia may also result in observable abnormalities in electrocardiogram readings.^9^, ^17^

Bedside ultrasound is a valuable initial tool for assessing patients with nonspecific abdominopelvic complaints. It helps evaluate both anterior and posterior vesicular areas, bladder wall irregularities, and pelvic landmarks.^8^, ^18^ The combined abdominopelvic CT exam is the preferred modality for nonspecific complaints. Conventional abdominal noncontrast CT has an accuracy rate of only 60.6% for bladder injury compared to 95.9% for the retrograde cystogram. Exploratory laparotomy is the gold standard for diagnosis, with most cases diagnosed intraoperatively.^19^ CT cystography is recommended for preoperative evaluation of suspected bladder rupture, allowing simultaneous assessment of multiple abdominal organs.^14^

While there's no specific guideline for SRUB, both the European Association of Urology and The American Urological Association concur that intraperitoneal bladder ruptures necessitate surgical repair. For uncomplicated extraperitoneal bladder injuries, conservative treatment is an option. However, surgical repair is recommended for complex extraperitoneal bladder injuries and all intraperitoneal ruptures, and usually involves either open or laparoscopic cystorrhaphy. In open cystorrhaphy, a lower abdominal incision is made to directly access the bladder, where the tear is trimmed and carefully sutured in multiple layers for a secure closure. Laparoscopic cystorrhaphy, on the other hand, employs minimally invasive techniques with small incisions and the assistance of a laparoscope and specialized tools to repair the rupture. Following surgery, patients receive pain relief, antibiotics, and urinary catheterization for bladder drainage, with close monitoring during follow‐up appointments to ensure proper healing. The choice between open and laparoscopic methods depends on factors such as the severity of the injury, patient characteristics, and surgeon experience.^8^, ^20^, ^21^

## 4 CONCLUSION

This case report highlights the rarity and complexity of SRUB, particularly in patients with a history of alcohol abuse. Despite its low incidence, the high mortality rate underscores the importance of early diagnosis and appropriate treatment. The varied and non‐specific presentations of SRUB pose significant diagnostic challenges, often leading to misdiagnosis. This case underscores the need for a high index of suspicion and comprehensive patient history, especially in cases involving recent heavy alcohol consumption. Further research and specific guidelines for SRUB could enhance patient prognosis and outcomes.

## AUTHOR CONTRIBUTIONS

**Shritik Devkota:** Conceptualization; data curation; methodology; writing – original draft. **Sugat Adhikari:** Conceptualization; data curation; methodology; writing – original draft. **Manbir Singh:** Conceptualization; data curation; investigation; supervision; writing – original draft. **Samiksha Lamichhane:** Writing – original draft. **Dipendra Adhikari:** Writing – original draft. **Bishal Koirala:** Writing – original draft. **Harsha Bhola:** Conceptualization; supervision; writing – original draft.

## FUNDING INFORMATION

The authors declare that they have no known competing financial interests or personal relationships that could have appeared to influence the work reported in this paper.

## CONFLICT OF INTEREST STATEMENT

The authors have declared that no competing interests exist.

## ETHICS STATEMENT

The authors declare that the procedures were followed according to the regulations established by Clinical Research and Ethics Committee and to the Helsinki Declaration of the World Medical Association updated in 2013.

## CONSENT

Written informed consent was obtained from the patient to publish this report in accordance with the journal's patient consent policy.

## Data Availability

All the necessary clinical images and case details are already provided within the manuscript along with comprehensive literature sources.
